# Condom use among long-term intimate partners using drugs baseline results from a randomized trial in Ukraine

**DOI:** 10.1186/1742-4690-9-S1-P109

**Published:** 2012-05-25

**Authors:** Liudmyla Shulga, Tatiana Andreeva

**Affiliations:** 1 ICF International HIV AIDS Alliance in Ukraine, Kiev, Ukraine; 2 National University of Kyiv Mohyla Academy, Ukraine

## Introduction

Ukraine faced changes in HIV transmission routes and in 2007 sexual transmission exceeded parenteral way. In response to situation change couple’s counseling for IDUs was introduced. Data presented in the abstract are part of the randomized trial to test couple’s counseling as a mean of HIV prevention among IDUs.

## Methods

Baseline data were collected in June - September 2011. 560 IDU couples in 10 cities were surveyed and screened for HIV and Hepatitis C. Participants were recruited using respondent-driven sampling method.

## Results

Age of respondents varied between 30 and 33 years old with women being about 3 years younger compared to men. On average, duration of relationship was 3.7 years. Only 15% of respondents were married, while majority (64.9%) lived in a civil marriage; 20% declared having close intimate relationships. At the same time women tended to consider living together as “marriage” while men differentiated between these two categories. Men had regular jobs more often compared to women (23.8 vs 15.8%), as well as odd jobs (53% vs. 31% respectively). Data showed that there was no difference observed between men and women in sexual activity with regular partners. But men reported having more occasional sexual partners (3 partners/30 days), and women were more often engaged into commercial sex (19 partners/30 days). Condom use with occasional (80%) and commercial (88%) sex partners took place more often compared to sexual intercourse with regular partners (44%). Condom use during anal and oral intercourse was much lower and varied between 8% with regular partners and 25% with occasional partners. The most common reasons for avoiding condom use were sensitivity decrease (28%).

## Conclusions

Intimate partners are often not engaged into sexual relationships, even if they live together; drug use is sees as a more intimate process. Sex education for IDUs should account for nature of intimate relationsips; focus on reasons of condom non-use; and promote protected anal and oral intercourses. Education should also teach to understand risks related to own or partner's extramarital sex.

